# Deciphering the Population Characteristics of Leiqiong Cattle Using Whole-Genome Sequencing Data

**DOI:** 10.3390/ani15030342

**Published:** 2025-01-24

**Authors:** Yingwei Guo, Zhihui Zhao, Fei Ge, Haibin Yu, Chenxiao Lyu, Yuxin Liu, Junya Li, Yan Chen

**Affiliations:** 1 State Key Laboratory of Animal Biotech Breeding, Institute of Animal Science, Chinese Academy of Agricultural Sciences (CAAS), Beijing 100193, China; yingwei.guo@foxmail.com (Y.G.); gefei@caas.cn (F.G.); qinqiqwer@163.com (C.L.); yuxinliu22@163.com (Y.L.); 2 College of Coastal Agricultural Sciences, Guangdong Ocean University, Zhanjiang 524088, China; zhzhao@gdou.edu.cn (Z.Z.); yuhb@gdou.edu.cn (H.Y.); 3 Institute of Animal Husbandry and Veterinary Science, Tianjin Academy of Agricultural Sciences, Tianjin 300381, China

**Keywords:** population genetics, genomic characteristics, Leiqiong cattle, selective sweep

## Abstract

Long-term geographic isolation and breeding programs both influence population characteristics. Leiqiong cattle, a native breed from the southernmost region of China, are renowned for disease and heat resistance, with two subgroups on Hainan Island and the Leizhou Peninsula. However, the genomic differences between them remain unexplored. In this study, we conducted genomic comparisons using whole-genome sequencing data from the two subgroups of Leiqiong cattle and three commercial breeds to assess their population structures. Leiqiong cattle in Hainan exhibited lower genetic diversity and a pure ancestral content due to their isolation from the mainland. In contrast, the subgroup in Guangdong displayed higher genetic diversity and mixed ancestry, influenced by the intrusion of commercial breeds. The genetic divergence between them was evaluated by estimating a genetic distance of 0.08 and a split time of 3400 to 4250 years ago, highlighting the role of geographical barriers in speciation. Notably, two candidate genes were identified through selection sweeps, including *PIP4K2A*, potentially related to immunity, and *TNFSF4*, possibly involved in hair follicle development. Our findings reveal the different genetic structures and genomic characteristics in the two subgroups of Leiqiong cattle, providing valuable insights into their evolutionary history and establishing a foundation for future breeding strategies.

## Simple Summary

Leiqiong cattle, native to China’s southernmost region, are prized for resisting disease and enduring heat. However, two distinct subgroups of these cattle—one in Hainan and the other in Guangdong—have evolved differently due to their separate environments. Our research utilized whole-genome sequencing data to investigate how these cattle have genetically diverged from each other and other commercial cattle breeds. We found that the Hainan subgroup has preserved a purer ancestry and exhibits less genetic variation, likely due to its isolated location. In contrast, the Guangdong subgroup shows greater genetic diversity and evidence of mixing with commercial breeds, likely resulting from historical crossbreeding. Our study identified genes that may account for the cattle’s disease resistance and other adaptive traits. Understanding these genetic differences enhances our appreciation of the impact of breeding practices and geographic isolation on livestock. This knowledge not only aids in conserving unique genetic traits but also informs breeding strategies to ensure the sustainability and resilience of cattle breeds in changing environments.

## 1. Introduction

Cattle are one of the earliest domesticated animals in the world [1]. By providing milk, meat, leather, and other products, they have become an indispensable part of human society and have spread across the globe along with human migration and trade [2]. In the long process of natural and artificial selection, cattle have formed various breeds with distinct characteristics. In China, there are 55 indigenous breeds of cattle (*Bos taurus*) adapted to different geographical and climatic environments. However, due to continuous hybridization with commercial breeds for genetic improvement, the genetic diversity and population size of Chinese local breeds are under threat [3,4]. Additionally, due to long-term geographical isolation and human captivity, distinct differences have emerged among subgroups of the same breed in different regions, even though they share the same name. It is crucial to assess their genetic diversity and population structure to prevent further genetic loss and admixture in these Chinese indigenous cattle breeds.

Leiqiong cattle, a superior cattle breed from the subtropics of southern China, are found in Guangdong Province and Hainan Province. At present, there are about 225,000 Leiqiong cattle in China, including 15,000 in Guangdong Province and 210,000 in Hainan Province. They are known for their heat tolerance, ability to thrive on rough feed, and disease resistance [5]. The subgroup in Guangdong is mainly distributed in the Leizhou Peninsula, the southernmost region of Guangdong Province. With the development of agricultural cultivation technology and the popularization of agricultural mechanization, Leiqiong cattle in Guangdong have shifted from being primarily draft animals to meat production animals. However, their small size, slow growth rate, and poor meat yield result in lower economic returns compared to commercial breeds. To enhance growth performance and meat quality, many farms have crossbred Leiqiong cattle with commercial breeds like Limousin, South Devon, and Angus to utilize their resource advantages better. Unfortunately, inadequate management practices and the absence of a comprehensive pedigree registration system have led to uncontrolled crossbreeding with commercial breeds. This has resulted in confused pedigrees among hybrid offspring and reduced heterosis [6]. The other subgroup of Leiqiong cattle resides on Hainan Island, a hot and rainy climate with an average annual temperature of 24.5 °C and precipitation ranging from 1600 to 2000 mm. Through long-term adaptation to Hainan’s hot and humid environment, Leiqiong cattle in Hainan have developed unique traits that enhance their resistance to heat and piroplasmosis [5]. Although the two subgroups are very close geographically, the Qiongzhou Strait between Guangdong Province and Hainan Province serves as a natural geographical barrier, making it challenging to have gene flow between the two populations of Leiqiong cattle [7]. They have adapted to their respective habitats under independent natural environments and artificial selection. The extent of genomic differences between the two subgroups remains unclear. Therefore, it is worth exploring whether the two subpopulations of Leiqiong cattle have differentiated at the genomic level.

Although bead chips have been widely used in population genetics [3,8,9,10], their results are limited by low marker density. In particular, when performing selective sweep analysis, many methods rely on accurate linkage disequilibrium (LD) information [11,12]. However, even high-density microarrays struggle to assess LD levels in a population comprehensively. High-throughput sequencing captures all variations on the whole genome. This approach allows researchers to analyze population structures, environmental adaptations, and evolutionary mechanisms [13]. As sequencing technologies advance and more reference genome sequences become available from public databases, whole genome resequencing has become a valuable tool for animal breeding and population genetics. Several studies have explored the genetic diversities and population structures of sheep [14,15], goats [16], pigs [17], chicken [18,19], and different populations of cattle [4,20,21,22,23] using next-generation sequencing. However, to our knowledge, the genetic diversity and population structure of Leiqiong cattle have not been investigated.

To understand the population characteristics of Leiqiong cattle and the genomic differences between the subpopulations in Guangdong and Hainan, we collected and analyzed whole-genome sequencing data from 241 cattle (including Angus, Limousin, SouthDevon, Leiqiong cattle in Guangdong, and Leiqiong cattle in Hainan). Our results offer new insights into the genetic diversities, population structures, divergence time, and selective sweeps of Leiqiong cattle in Guangdong and Hainan compared to other breeds.

## 2. Materials and Methods

### 2.1. Ethics Statement

All animals were treated following the guidelines for experimental animals, which the Council of China established. Animal experiments were approved by the Science Research Department of the Institute of Animal Science, Chinese Academy of Agricultural Sciences (CAAS) (Beijing, China).

### 2.2. Sample Collection and Genome Sequencing

To represent the overall genetic diversity of Leiqiong cattle, we collected 41 samples from Zhanjiang City of Guangdong Province and 44 samples from Haikou City of Hainan Province. Genomic DNA was extracted from the blood samples of the animals using a standard phenol–chloroform protocol. For each individual, at least 5 mg genomic DNA was used to construct paired-end libraries with an insert size of 500 bp according to Illumina’s library preparation protocol. All paired-end reads were generated through the DNBSEQ-T7 sequencing platform from BGI in Beijing, China. Additionally, we collected 47 Angus, 50 Limousin, and 50 South Devon cattle from our other studies.

### 2.3. Read Mapping and SNP Calling

All clean reads were mapped to the *Bos taurus* latest reference genome (ARS-UCD2.0) using BWA-MEM (0.7.13-r1126) with default parameters [24]. Duplicate reads were removed using Picard Tools (http://broadinstitute.github.io/picard/, accessed on 23 December 2024). SNPs were detected by the Genome Analysis Toolkit (GATK, version 3.8-0-ge9d806836) [25].

HaplotypeCaller generated the GVCF file of each sample. The CombineGVCFs subcommand merged all GVCF files and GenotypeGVCFs performed joint genotyping. The HardFilter pipeline was used to filter low-quality variants with the parameter recommended by GATK best practices guidelines [25,26]: (1) SNPs with mean depth (for all samples) <1/3× and >3× (×, overall mean sequencing depth across all SNPs); (2) quality by depth, QD < 2.0; (3) phred-scaled variant quality score, QUAL < 30.0; (4) strand odds ratio, SOR > 3.0; (5) Fisher strand, FS > 60.0; (6) mapping quality, MQ < 40.0; (7) mapping quality rank sum test, MQRankSum < −12.5; and (8) read position rank sum test, ReadPosRankSum < −8.0. BCFtools [27] filtered out non-biallelic SNPs and SNPs with missing genotype rates >0.1 or minor allele frequency <0.01. After this, a total of 24,688,652 SNPs were retained.

The linkage disequilibrium (LD) prune analysis was performed using PLINK v1.9 [28] with the option “--indep-pairwise 50 5 0.4”. PLINK calculated the LD between each pair of SNPs in a window of 50 SNPs with a step size of 5 SNPs and removed one of a pair of SNPs if the LD was more significant than 0.4. Finally, a total of 2,314,258 SNPs were obtained.

### 2.4. Genome-Wide Patterns of Genetic Diversity and Divergence

The nucleotide diversity of each population was assessed using a sliding window approach (50 kb windows with 25 kb steps) with VCFtools [29]. Linkage disequilibrium (LD) was measured using the r2 statistic and calculated with PopLDDecay [30]. The size history of each population was estimated using SMC++ v1.15.2 [31]. A generation time of 4~5 years and a mutation rate of 1.2 × 10^−8^ mutations per nucleotide per generation were used [32].

### 2.5. Phylogenetic and Population Structure Analyses

A phylogenetic tree was constructed from the SNP data using the neighbor-joining method in the program RapidNJ [33]. Principle component analysis was carried out using the smartPCA program of the EIGENSOFT [34] package. Population structure was further inferred using ADMIXTURE [35] with kinship (K) set from 2 to 5 and visualized using PONG [36].

### 2.6. Selective Sweep Analysis

We screened the genomic regions under selection with the most significant differences in genetic diversity (*θπ*_(Leiqiong_Guangong)_/*θπ*_(Leiqiong_Hainan)_) and F_st_ outliers between Leiqiong_Guangdong and Leiqiong_Hainan using VCFtools [29]. We also performed XP-EHH analysis using the default settings of selscan v2.0.0 [37]. The π ratio, F_st_, and average normalized XPEHH score were calculated for 50 kb windows with 25 kb steps. The top 1% of windows were identified as significant genomic regions.

### 2.7. Functional Enrichment Analyses

Overlapping genes supported by at least two methods were defined as candidate genes for positive selection. Enrichment analysis of these candidate genes was enriched using the DAVID website [38] to explain their biological functions, and a *p* value <0.05 was considered to indicate significant enrichment.

## 3. Results

### 3.1. Whole-Genome Sequencing and Genetic Variation

We generated genomic data from 41 Leiqiong cattle in Guangdong and 44 Leiqiong cattle in Hainan. We collected another 147 cattle of three populations, including Angus, Limousin, and South Devon cattle, from our other studies. All clean reads were aligned to ARS-UCD2.0 with an average coverage rate of 99.79% (Table S1). The average depth of the reads was 16.58× (Table S1). After the filtering processes, a total of 24,688,652 biallelic SNPs were ultimately retained.

### 3.2. Population Structure and Relationships

**Figure 1:**
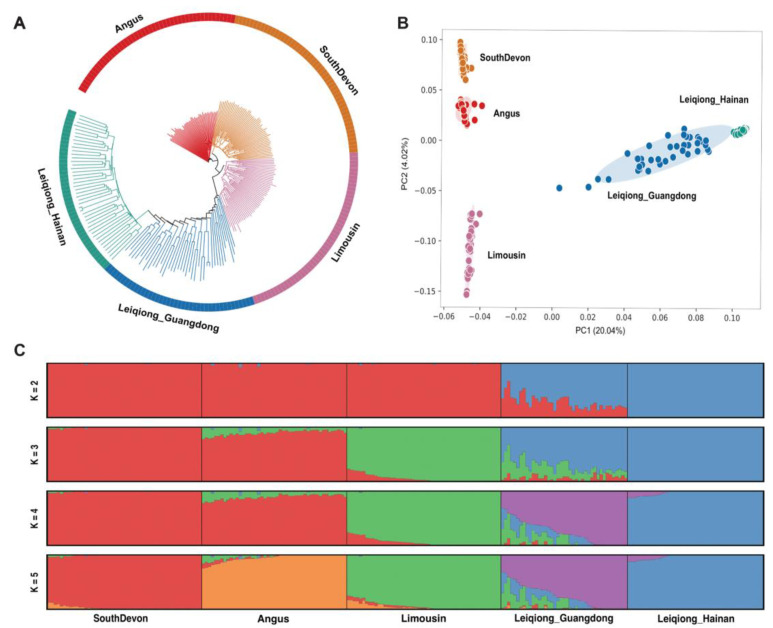
Population structure of Leiqiong cattle. (**A**) Neighbor-joining tree of the relationships between Leiqiong cattle and other cattle. (**B**) Principal component analysis. (**C**) Genetic structure using the ADMIXTURE program.

Based on the genomic SNPs data, the ADMIXTURE, Principal Component Analysis (PCA), and neighbor-joining (NJ) tree methods were used to explore the population differentiation and phylogenetic relationships between Leiqiong cattle and other breeds. The results from the NJ tree showed five distinct branches for the five populations. The two groups of Leiqiong cattle were closer to each other compared to the other breeds (Figure 1A). PCA revealed distinct geographical clustering among cattle populations (Figure 1B). The PC1 showed that the groups of Leiqiong cattle deviated from the three commercial breeds. We found that the two groups of Leiqiong cattle were also distinct from each other. It is worth mentioning that the distribution of Leiqiong cattle in Hainan was very concentrated, while the population in Guangdong was very dispersed, indicating that the population in Guangdong has higher diversity.

We used clustering models to predict ancestral populations and set K = 2 through K = 5 for ADMIXTURE analysis for all 241 samples (Figure 1C). When K = 2, the cattle breeds were genetically separated from Leiqiong cattle and other commercial breeds. When K = 3, we found that the Leiqiong cattle in Guangdong exhibited a mixed ancestral content of Limousin cattle and Leiqiong cattle in Hainan. This was consistent with the recent history of crossbreeding and improvement in the Leiqiong cattle of Guangdong. When K = 4, Leiqiong cattle in Guangdong and Hainan had different ancestral content. Leiqiong cattle in Guangdong shared only 11.1% of their ancestry with Leiqiong cattle in Hainan. When K = 5, 11.6% of the genomic ancestral content of Leiqiong cattle in Guangdong was still shared with Limousin cattle. However, Leiqiong cattle in Guangdong shared almost no ancestry with Angus (0.6%) or South Devon (0.8%) cattle.

### 3.3. Patterns of Genomic Variation

**Figure 2:**
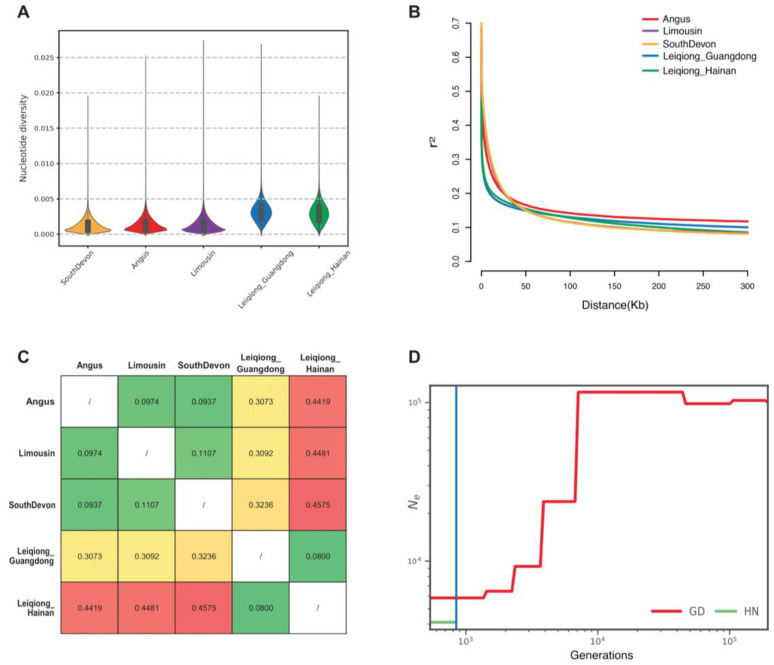
Genomic characteristics. (**A**) Box plots of nucleotide diversity in 50 kb sliding windows with 25 kb steps. (**B**) Decay of linkage disequilibrium (LD; r2) for each breed. (**C**) Genetic distance estimated by F_st_ values among breeds. (**D**) Divergence time between Leiqiong cattle in Guangdong and Hainan using SMC++.

The genetic variation patterns of the five cattle populations were analyzed by evaluating nucleotide diversity, genetic distance, LD, and split time. A higher nucleotide diversity of Leiqiong cattle indicated that they had greater genetic diversity than that of other commercial cattle (Figure 2A). Among Leiqiong cattle, the diversity of the subgroup in Guangdong (0.0032) exhibited slightly higher diversity than the subgroup in Hainan (0.0030). The average LD of all breeds decreased rapidly within 50 kb, among which, the LD of the two groups of Leiqiong cattle had the fastest decline. The average LD of Leiqiong cattle in Guangdong and Hainan were 0.10 and 0.08, respectively (Figure 2B). The genetic distances estimated by the F_st_ ranged from 0.08 to 0.46 between these populations (Figure 2C). The F_st_ values of Leiqiong cattle in Guangdong with Angus, Limousin, and South Devon were 0.31, 0.31, and 0.32, respectively. In contrast, the F_st_ values of Leiqiong cattle in Hainan with these breeds were 0.44, 0.45, and 0.46, respectively. The F_st_ values between Angus and Limousin, Angus and South Devon, and Limousin and South Devon were 0.10, 0.09, and 0.11, respectively. The mean genetic distance between the two groups of Leiqiong was 0.08, which was comparable to the values observed among the other three breeds. There was already a divergence between Leiqiong cattle in Guangdong and Hainan. Therefore, we used SMC++ to estimate the split time of the two subgroups (Figure 2D). The result indicated that Leiqiong cattle in Guangdong and Hainan diverged approximately 3400 to 4250 years ago (850 generations).

### 3.4. Genome-Wide Selective Sweep Between the Subgroup of Leiqiong Cattle

**Figure 3:**
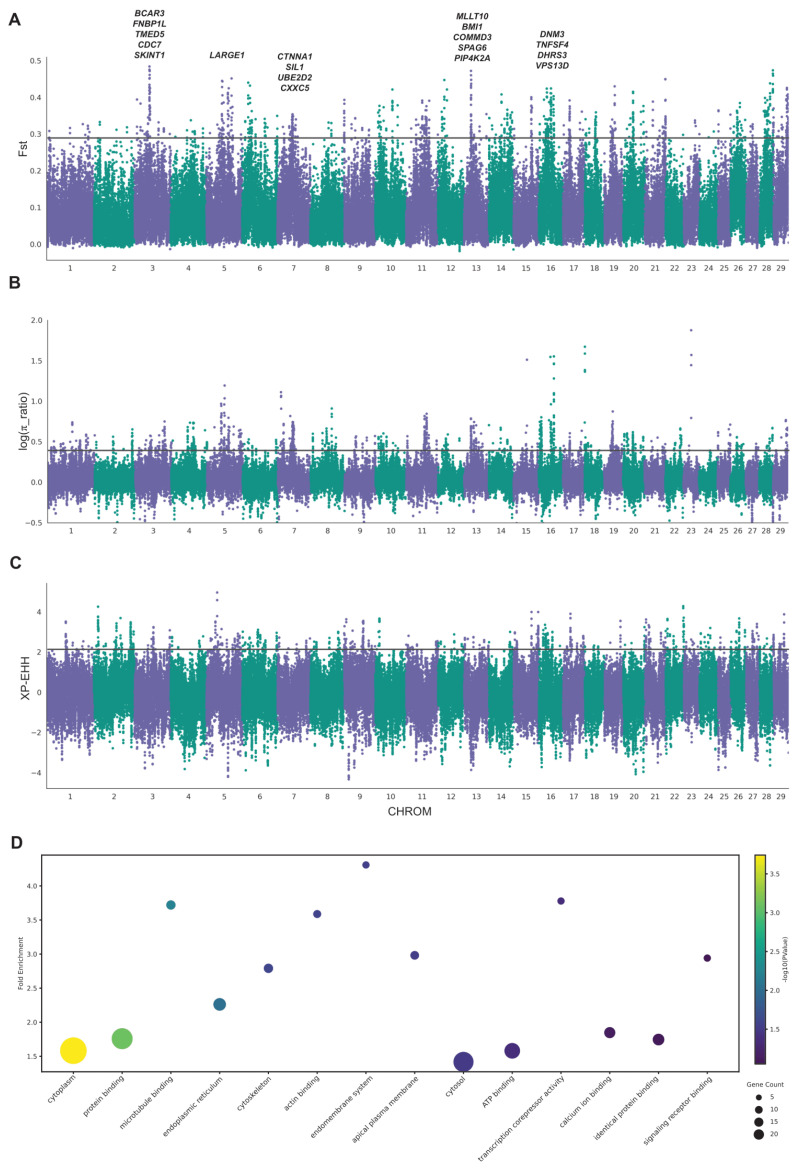
Analysis of selective sweeps in the genome of Leiqiong cattle. (**A**–**C**) Manhattan plot of selective sweeps in Leiqiong cattle using F_st_, *θπ* ratio, and XP-EHH, respectively. (**D**) The Kyoto Encyclopedia of Genes and Genomes pathways from the enrichment analysis of the candidate genes.

We employed three methods (F_st_, *θπ* ratio, and XP-EHH) to identify selective sweeps between Leiqiong cattle in Guangdong and Hainan (Figure 3A–C). Outlier regions (top 1%) identified by these methods were considered candidate regions for further analysis. The number of genes within the outlier windows was determined using the Fst, *θπ* ratio, and XP-EHH methods of 744, 768, and 630, respectively (Tables S2–S4). To avoid false positives, we considered the genes identified by at least two methods as the candidate genes. Finally, a total of 307 candidate genes under selection were identified (Figure S1; Table S5). Notably, the candidate genes were significantly overrepresented (*p* < 0.05) in several critical biological pathways, including cytoplasm, protein binding, microtubule binding, and endoplasmic reticulum (Figure 3D).

**Figure 4:**
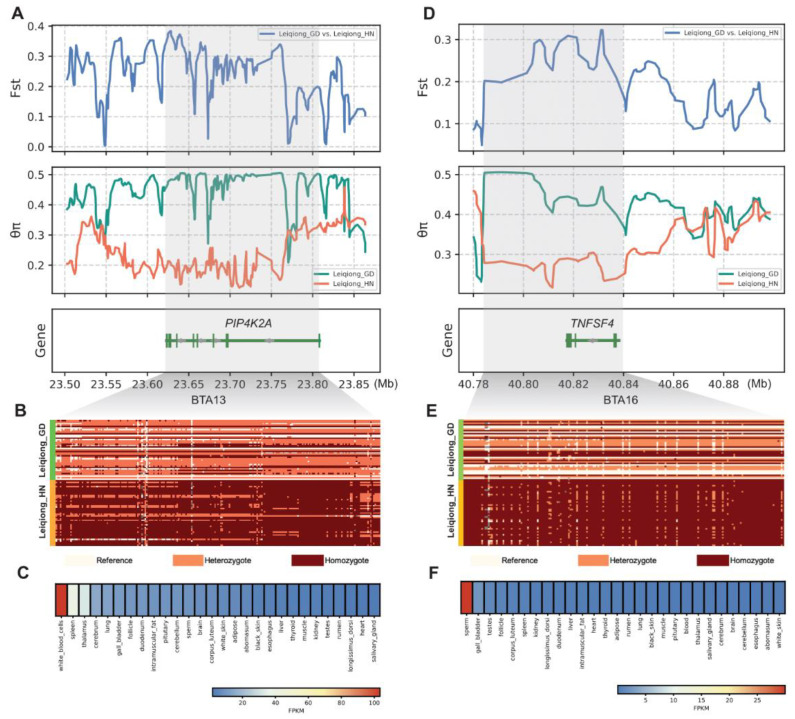
Analysis of the candidate genes in Leiqiong cattle. (**A**) Fixation index (F_st_) values and nucleotide diversity at the *PIP4K2A* gene region. (**B**) SNPs on the *PIP4K2A* gene were used to construct the haplotype heatmap. (**C**) Gene expression of *PIP4K2A* in different cattle tissues (http://animal.nwsuaf.edu.cn/code/index.php/RGD, accessed on 23 December 2024). (**D**) Fixation index (F_st_) values and nucleotide diversity at the *TNFSF4* gene region. (**E**) SNPs on the *TNFSF4* gene were used to construct the haplotype heatmap. (**F**) Gene expression of *TNFSF4* in different cattle tissues (http://animal.nwsuaf.edu.cn/code/index.php/RGD, accessed on 23 December 2024).

Among the numerous candidate genes, *PIP4K2A* exhibited notably high F_st_ values, low nucleotide diversity, and long haplotype patterns in Leiqiong cattle in Hainan (Figure 4A,B). *PIP4K2A* encodes the Phosphatidylinositol-5-Phosphate 4-Kinase Type 2 Alpha protein, associated with acute lymphoblastic leukemia susceptibility in humans [39]. Moreover, the Ruminant Genome Database (RGD) [40] showed that the expression levels of *PIP4K2A* in cattle were significantly higher in white blood cells than in other tissues (Figure 4C). Another region under selection was from 40.78 Mb to 40.84 Mb on BTA16, harboring the *TNFSF4* gene (Figure 4D,E). *TNFSF4* is a member of the tumor necrosis factor superfamily, regulating the proliferation, differentiation, and apoptosis of cells [41]. Other research has shown that *TNFSF4* plays a role in both the initiation and development of feather follicle morphogenesis [42]. Moreover, the expression levels of *TNFSF4* were significantly higher in sperm than in other tissues (Figure 4F).

We also screened out other candidate genes including *BCAR3*, *FNBP1L*, *TMED5*, *CDC7*, *SKINT1*, *LARGE1*, *CTNNA*, *MLLT10*, *BMI1*, *COMMD3*, *SPAG6*, *DNM3*, *DHRS3*, and *VPS13D* (Table S5). Therefore, these comparisons highlight crucial genes associated with genetic adaptation between Leiqiong cattle in Guangdong and Hainan.

## 4. Discussion

Analyzing population structure and genetic diversity is crucial for effectively using and conserving domestic animal genetic resources. Genomic variation has been observed in Chinese local cattle breeds, including Qinchuan cattle [43], Jiaxian Red cattle [21], and Anxi cattle [4]. Leiqiong cattle, an important breed from southern China, are renowned for their heat and disease resistance. However, few studies have examined the genetic structures and genomic characteristics of different populations of Leiqiong cattle. In this study, we collected samples from Leiqiong cattle and three other European breeds to explore the genetic diversities, population structures, divergence time, and selective sweeps of Leiqiong cattle in Guangdong and Hainan compared to other breeds.

The Leiqiong cattle in the Hainan subgroup are isolated on Hainan Island, limiting genetic admixture with other breeds. In contrast, Leiqiong cattle in Guangdong reside on China’s southern mainland, separated from Hainan Island by the Qiongzhou Strait, and have recently interbred with commercial breeds such as Angus and Limousin [6]. Different geographical environments and breeding programs result in distinct natural and artificial selection pressures for the two subgroups, leading to different population structures and genetic features between the two subgroups. Additionally, the phylogenetic tree showed that the five populations were divided into five independent clades and that the two populations of Leiqiong cattle were clustered closer than other populations. PCA showed similar results to those of the phylogenetic tree. However, the Leiqiong cattle in Hainan showed a more concentrated distribution than the subgroup in Guangdong, indicating that the population in Hainan was more homogeneous than the Leiqiong cattle in Guangdong. The admixture also showed that the Leiqiong cattle in Hainan had a pure ancestral composition from K = 2 to K = 5. In contrast, the Leiqiong cattle in Guangdong had a genomic breed composition (GBC) consistent with Limousin cattle when K = 3, 4, and 5 (Figure 1C). This aligns with the historical crossbreeding of Leiqiong cattle in Guangdong with Limousin cattle. As a common practice, local and commercial breeds are typically crossed and then backcrossed and selected to maintain the characteristics of the local breeds [44]. As the backcrossing is carried out, the genetic segments of the commercial varieties are gradually diluted. This explains the varying proportions of Limousin composition found in different Leiqiong cattle in Guangdong (Figure 1C). It is worth mentioning that previous studies indicated that the Leiqiong cattle in Guangdong may also have admixture with Angus and South Devon cattle [6], but no evidence of their hybridization was observed in our samples.

Compared to commercial breeds, Leiqiong cattle demonstrated greater nucleotide diversity and reduced linkage disequilibrium (LD), suggesting a shorter history of artificial selection and greater genetic potential. In comparison, the commercial breeds showed lower genetic diversities and higher LD due to extensive artificial selection. The samples of these commercial breeds were also collected in China, and their Funder Effect may also have resulted in lower genetic diversities. The separation of two subgroups of Leiqiong is also reflected by the genome characteristics. The genetic distance between the two groups of Leiqiong cattle was 0.080. The distance between Angus vs. Limousin and Angus vs. South Devon cattle was only 0.097 and 0.094, respectively. The F_st_ between them and the F_st_ between the two populations of Leiqiong were at the same level. Therefore, genetic divergence between the two subgroups of Leiqiong cattle has been formed, although they both belong to the Leiqiong breed. We thus applied SMC++ to estimate their split time [31], which incorporated information from the site frequency spectrum, as estimated from a larger number of unphased genomes, enabling the inference of effective population sizes into more recent periods [45]. However, it should be noted that the estimated divergence time for two subgroups may have been inflated due to the crossbreeding of Leiqiong cattle in Guangdong with other breeds.

Many methods have been developed to detect the selective sweeps on the genome [46]. Here, we evaluated signals of selection between the subgroups of the Leiqiong cattle genome based on nucleotide diversity (*θπ*), fixation index (F_st_), and cross-population extended haplotype-based homozygosity (XP-EHH). It is important to note that hybridization between commercial and local breeds has altered the genetic background of local breeds, diminishing the adaptive signals shaped initially by natural selection. Despite not excluding the strongly mixed regions from our analysis, we utilized whole-genome sequencing data to enhance our ability to distinguish between selective sweeps and mixed signals.

In total, we identified 307 candidate genes, and further enrichment analysis indicated a significant overrepresentation of genes associated with the cytoplasm, protein binding, microtubule binding, and endoplasmic reticulum. Considering the long-term survival environment of Leiqiong cattle, as well as the high temperatures and heavy rain, these pathways may explain the heat and disease resistance traits of Leiqiong cattle. Among these genes, we identified *PIP4K2A* and *TNFSF4* as key candidate genes potentially linked to immunity and hair follicle development. *PIP4K2A*, the precursor to second messengers of the phosphoinositide signal transduction pathways, is thought to regulate secretion and cell proliferation, differentiation, and motility [47,48,49]. A human study demonstrated that the depletion of *PIP4K2A/B* inhibited tumor growth, both in vitro and in vivo, selectively in p53-deficient tumors [50]. Previous studies found the *PIP4K2A* was associated with meat quality in pigs [51,52]. Proteomic analysis of the hypothalamus and pituitary gland in Brahman cattle suggests that *PIP4K2A* might be an essential driver of puberty in cattle [53]. Another previous study in Xiangxi cattle identified this gene as being related to meat quality [54]. In this study, the lower genetic diversity of the *PIP4K2A* in Leiqiong cattle in Hainan indicated a strong selection of this gene within this subgroup. A humid and hot environment often leads to animals falling ill, but Leiqiong cattle have shown resistance to parasites and piroplasmosis [5]. A high expression level of *PIP4K2A* was observed in white blood cells, indicating its potential role in enhancing disease resistance in Leiqiong cattle. However, further experimental studies are required to confirm its function in Leiqiong cattle. The *TNFSF4* gene encodes the tumor necrosis factor (TNF) ligand family cytokine. This protein plays a critical role in interactions between T cells and antigen-presenting cells (APCs) and facilitates the adhesion of activated T cells to endothelial cells. Members of the TNF superfamily regulate cellular processes such as proliferation, survival, or apoptosis [41]. In humans, the *TNFSF4* has been linked to systemic lupus erythematosus [55,56]. A polymorphism in the promoter region of the *TNFSF4* gene has been demonstrated to be associated with the risk of myocardial infarction [57]. It also affects atherosclerosis susceptibility in mice [58]. In our study, the *TNFSF4* gene exhibited high expression levels in sperm cells, suggesting a potential association with reproductive traits in Leiqiong. Further validation is necessary to confirm this observation. Other studies showed that the *TNFSF4* gene was also related to hair follicle development [42]. Previous studies found that the *TNFSF4* gene was associated with wool traits in Chinese sheep [59] and Merino sheep [60]. Reducing hair density to enhance heat dissipation is a typical temperature regulation strategy for animals [61,62]. Leiqiong cattle have lived in tropical areas for a long time, and this gene may enhance the heat resistance of Leiqiong cattle by regulating the development of hair follicles. Moreover, these two genes also exhibited different haplotype patterns and high F_st_ values between Leiqiong cattle in Guangdong and Hainan, suggesting different selection pressures in the two subgroups of Leiqiong cattle due to different environments and management practices.

## 5. Conclusions

This study has unveiled the genomic landscape of Leiqiong cattle, shedding light on the genetic divergence between the two geographically distinct subgroups from Hainan Island and the Leizhou Peninsula. Additionally, we identified a series of candidate genes likely to play pivotal roles in adaptation to specific environments, encompassing aspects of immunity and hair follicle development. Our findings provided a new paradigm for understanding how geographic isolation and hybrid improvement affect population structure and adaptive evolution in specific environments. Future research should extend the current findings in depth to elucidate the regulatory mechanisms of the identified candidate genes and their contribution to environmental adaptation, ultimately informing sustainable breeding practices that balance genetic improvement with the conservation of genetic heritage in Leiqiong cattle.

## Data Availability

The data presented in this study are available upon request from the corresponding author. The data are not publicly available for privacy reasons.

## Supplementary Materials

The following supporting information can be downloaded at https://www.mdpi.com/article/10.3390/ani15030342/s1: Table S1. Sample information in this study. Table S2. Gene annotation of putative regions under selection based on the top 1% value of global F_st_. Table S3. Gene annotation of putative regions under selection based on the top 1% value of π_ratio. Table S4. Gene annotation of putative regions under selection based on the top 1% value of XP-EHH. Table S5. Gene annotation of the overlapped genes identified by at least two methods. Figure S1. Venn diagram showing the gene overlap among F_st_, π_ratio, and XP-EHH.
